# Deficiency of Soluble Epoxide Hydrolase Protects Cardiac Function Impaired by LPS-Induced Acute Inflammation

**DOI:** 10.3389/fphar.2018.01572

**Published:** 2019-01-14

**Authors:** Victor Samokhvalov, K. Lockhart Jamieson, Ahmed M. Darwesh, Hedieh Keshavarz-Bahaghighat, Tim Y. T. Lee, Matthew Edin, Fred Lih, Darryl C. Zeldin, John M. Seubert

**Affiliations:** ^1^Faculty of Pharmacy and Pharmaceutical Sciences, University of Alberta, Edmonton, AB, Canada; ^2^Department of Pharmacology, Faculty of Medicine, University of Alberta, Edmonton, AB, Canada; ^3^Division of Intramural Research, National Institute of Environmental Health Sciences, National Institutes of Health, Research Triangle Park, NC, United States

**Keywords:** soluble epoxide hydrolase, cardiac function, mitochondrial function, LPS, inflammation

## Abstract

Lipopolysaccharide (LPS) is a bacterial wall endotoxin producing many pathophysiological conditions including myocardial inflammation leading to cardiotoxicity. Linoleic acid (18:2n6, LA) is an essential n-6 PUFA which is converted to arachidonic acid (20:4n6, AA) by desaturation and elongation via enzyme systems within the body. Biological transformation of PUFA through CYP-mediated hydroxylation, epoxidation, and allylic oxidation produces lipid mediators, which may be subsequently hydrolyzed to corresponding diol metabolites by soluble epoxide hydrolase (sEH). In the current study, we investigate whether inhibition of sEH, which alters the PUFA metabolite profile, can influence LPS induced cardiotoxicity and mitochondrial function. Our data demonstrate that deletion of soluble epoxide hydrolase provides protective effects against LPS-induced cardiotoxicity by maintaining mitochondrial function. There was a marked alteration in the cardiac metabolite profile with notable increases in sEH-derived vicinal diols, 9,10- and 12,13-dihydroxyoctadecenoic acid (DiHOME) in WT hearts following LPS administration, which was absent in sEH null mice. We found that DiHOMEs triggered pronounced mitochondrial structural abnormalities, which also contributed to the development of extensive mitochondrial dysfunction in cardiac cells. Accumulation of DiHOMEs may represent an intermediate mechanism through which LPS-induced acute inflammation triggers deleterious alterations in the myocardium *in vivo* and cardiac cells *in vitro*. This study reveals novel research exploring the contribution of DiHOMEs in the progression of adverse inflammatory responses toward cardiac function *in vitro* and *in vivo.*

## Introduction

Exposure to the bacterial endotoxin lipopolysaccharide (LPS), a major component of the cell wall from Gram-negative bacteria, can trigger acute systemic reactions potentially leading to multiple organ failure (Lew et al., 2013). Prevailing theories attribute these failures to uncontrolled inflammatory responses that produce numerous deleterious effects such as extensive organelle dysfunction and ultimately cell death (Lew, 2003; Kozlov et al., 2011; Lew et al., 2013). Cardiac dysfunction is a common outcome following such acute inflammatory responses, which can ultimately lead to heart failure and subsequent death (Chagnon et al., 2005). Binding of LPS to TLR-4 receptors initiates the IKK-NF-kB inflammatory program releasing pro-inflammatory cytokines such as TNFα, IL-1, IL-6, and MCP-1 (Frantz et al., 1999; Chagnon et al., 2005; Charalambous et al., 2007). The heart represents a particularly vulnerable target for LPS as terminally differentiated cardiomyocytes abundantly express TLR4 receptors (Kozlov et al., 2011). Furthermore, significant amounts of energy are required to sustain proper cardiac contractile function, making the heart the major consumer of energy in the body on a weight basis. During heart failure, the myocardium has low ATP content due to a decreased ability to generate ATP by oxidative metabolism thus reducing contractile work (Brealey et al., 2002). Although the precise mechanisms by which adverse acute inflammatory reactions lead to organ failure are not clear, evidence suggests mitochondrial dysfunction is an important cause in the progression of cardiac failure (Frantz et al., 1999; Callahan and Supinski, 2005; Anderson et al., 2007).

Dietary sources of n-6 polyunsaturated fatty acids (n-6 PUFA) may be obtained from liquid vegetable oils, including soybean, corn, sunflower, safflower, and cottonseed oils. Linoleic acid (18:2n6, LA) is the primary source of the essential n-6 PUFA, which is converted to arachidonic acid (20:4n6, AA) by desaturation and elongation via enzyme systems within the body (Jamieson et al., 2017a). N-6 PUFA are metabolized into a plethora of bioactive eicosanoids through three primary enzymatic systems: cyclooxygenases (COX), lipoxygenases (LOX), and cytochrome P450 (CYP) enzymes (Mabalirajan et al., 2013). CYP epoxygenases generate linoleic epoxides including 9,10-epoxyoctadecenoic acid (9,10-EpOME) and 12,13-epoxyoctadecenoic acid (12,13-EpOME), which are further metabolized by soluble epoxide hydrolase (sEH) to form the corresponding linoleic diols 9,10-dihydroxyoctadecenoic acid (9,10-DiHOME) and 12,13-dihydroxyoctadecenoic acid (12,13-DiHOME) (Mabalirajan et al., 2013; Lynes et al., 2017). The eicosanoids produced act as potent lipid mediators that regulate cellular homeostasis, including inflammatory responses and cytotoxicity (Dennis and Norris, 2015; Wagner et al., 2017). Evidence has indicated many of the cytotoxic effects attributed to EpOMEs are in fact caused by their secondary metabolites DiHOMEs, which are formed in the reaction catalyzed by sEH (Moghaddam et al., 1997; Fleming, 2014). For example, loss of cardioprotection observed in transgenic mice overexpressing CYP2J2 in cardiomyocytes was related to an age-related increase accumulation of cardiotoxic derivatives such as DiHOMEs, which were reversed following inhibition of sEH (Chaudhary S. et al., 2013). Similarly, well documented evidence demonstrates that evoking anti-inflammatory responses can be achieved by inhibiting or deleting sEH (Schmelzer et al., 2005; Wagner et al., 2017). However, the biological effects and potency of many of the eicosanoid molecules remains largely unknown and yet to be identified in the context of orchestrating inflammatory responses in cardiovascular system.

In this study, we investigated the influence of sEH over LPS-induced acute inflammation responses and the involvement of CYP-derived metabolites of LA regarding cardiac and mitochondrial dysfunction and cellular injury. Our major findings suggest increased levels of DiHOMEs correlate with LPS-induced cardiac toxicity and markedly reduces mitochondrial function. In addition, exposure of cardiac cells to DiHOMEs *in vitro* resulted in development of cellular injury leading to cell death.

## Materials and Methods

### Animals

A colony of mice with targeted disruption of the *Ephx2* gene (sEH null) and wild-type littermates are maintained at the University of Alberta. All studies were carried out using male mice that were 2–3 months old and weighed 20–30 g. Mice received a single injection of LPS administered intraperitoneally (10 mg/kg) and were sacrificed 24 h later and samples collected. The dose of LPS injection was determined from previous myocardial studies and is considered a moderate dose (Lew et al., 2013; Strand et al., 2015). Body temperature was determined by measuring rectal temperature in animals using a digital thermometer (MT-Esatherm Ltd., 8172, Czechia) with a 2 mm sensor diameter. All animal experimental protocols were approved by the University of Alberta Health Sciences Welfare Committee and were performed in strict adherence to the guidelines set by the Canadian Council of Animal Care.

### Blood Glucose Monitoring

The level of blood glucose was determined using a calibrated glucometer OneTouch Ultra2 from (LifeScan Inc., Switzerland). The assay was performed in accordance to guidance manufacturer instruction.

### Mitochondrial Function

Hearts were ground with a mortar and pestle on dry ice and then homogenized in ice-cold buffer (20 mM Tris–HCl, 50 mM NaCl, 50 mM NaF, 5 mM sodium pyrophosphate, with 0.25 M sucrose added on the day of the experiment). Samples were centrifuged at 600 ×*g* for 10 min at 4°C to remove debris. Supernatant was collected and the protein content was measured by standard Bradford assay. The activities of citrate synthase (CS), NADH:ubiquinone oxidoreductase (complex I) and succinate dehydrogenase (SDH, complex II) were assessed spectrophotometrically as previously described (Akhnokh et al., 2016). Mitochondrial respiration was analyzed in freshly isolated cardiac mitochondria as described (Gedik et al., 2013). Briefly, heart homogenate was first centrifuged at 700 ×*g* for 10 min followed by centrifuging the supernatant at 10,000 ×*g* for 10 min, then the pellet was resuspended and washed using isolation buffer at 10,000 ×*g* for 10 min. Mitochondrial oxygen consumption was measured in isolated mitochondria (50 μg of mitochondrial protein) added to a chamber connected to OXYGRAPH PLUS system (Hansatech Instruments Ltd., Norfolk, England). Respiration rates were measured at 30°C in 2 ml of respiration buffer. Basal respiration was recorded after the addition of 5 mM malate and 10 mM glutamate as respiratory substrates for basal oxidative respiration. ADP-stimulated respiration was initiated by addition of 1 mM ADP then recorded. The efficiency of coupled oxidative phosphorylation was calculated and expressed as the ratio between basal and ADP-stimulated respiration rates.

ATP content was assessed using a colorimetrical assay kit (ab83355, Abcam Inc., Toronto, ON, Canada). Heart powders were homogenized and centrifuged at 15000 × *g* for 2 min and the resultant supernatant was assessed for ATP content. Standard curve for ATP and reaction mixture were prepared according to the kit manual in a 96-well plate and optical density (OD) was measured at 570 nm.

### Immunoblotting

Subcellular fractions isolated from hearts were subjected to western blot analysis as previously described (Darwesh et al., 2018; Endo et al., 2018). Briefly, 25 μg of protein from heart tissues was resolved on a 12% SDS-polyacrylamide gel and transferred to polyvinylidene difluoride membranes. Membranes were incubated with primary antibodies to sEH (1:1000, Santa Cruz Biotech, Inc., Cat No. sc22344), eNOS (1:500, Abcam, Toronto, ON, Cat No: ab76198), TLR4 (1:500, Abcam, Toronto, ON, Cat No: ab13556), succinate dehydrogenase A (SDH-A) (1:500, Cell Signaling Tech., Inc., Whitby, ON, Cat No. cs5839), CS (1:000, Abcam, Toronto, ON, Cat No: ab129095), cytochrome c oxidase subunit 1 (COX IV) (1:1,000, Cell Signaling Tech., Inc., Whitby, ON, Cat No: 4850), prohibitin (1:1000, Cell Signaling Technology, Inc., MA, Cat. NO: 2426), or GAPDH (1:5000, Cell Signaling Tech., Inc., Whitby, ON, Cat No: 5174S), secondary antibodies were used as 1:5000 dilution and expression was visualized with ECL reagent. Band intensities were expressed as fold change relative to control using Image J software (NIH, United States).

### Aconitase, 20S Proteasome and Malondialdehyde Assays

Aconitase activity was used as a marker of oxidative mitochondrial damage and evaluated using an ELISA kit (Abcam, Toronto, ON, Canada). 20S proteasome activity was used as a marker of unspecific degenerative processes and determined in tissue lysates based on monitoring the release of AMC by proteolytic cleavage of the peptide Suc-LLVY- AMC (CHEMICON Inc., Billerica, MA, United States) by 20S proteasomes. Fluorescence was monitored at wavelengths of 380 nm (excitation) and 460 nm (emission). Specific activity was determined from a standard curve established with AMC. The level of MDA was used as an overall marker of lipid peroxidation and assessed in reaction with thiobarbituric acid using a kit from (Abcam, Toronto, ON, Canada). The MDA-TBA adduct was quantified colorimetrically at 540 nm wavelength.

### LC – MS/MS Oxylipid Analysis

Tissue samples were stored at -80°C until processing. LC-MS/MS methods and multiple reaction monitoring have been adapted from previously established protocols (Newman et al., 2002; Edin et al., 2011) Samples were homogenized in 5 × volume of 0.1% acetic acid in 5% methanol containing 1 uM *trans-*4-[4-(3-adamantan-1-yl-ureido)-cyclohexyloxy]-benzoic acid (tAUCB), spiked with 3 ng PGE 2 -d9, d11-11,12-DHET, d11-11,12-EET (Cayman) as internal standards, extracted by liquid:liquid extraction with 3 ml ethyl acetate and dried in vacuum centrifuge. Samples were analyzed in duplicate 10 μL injections. Online liquid chromatography of extracted samples was performed with an Agilent 1200 Series capillary HPLC (Agilent Technologies, Santa Clara, CA, United States). Separations were achieved using a Halo C18 column (2.7 mm, 10062.1 mm; MAC-MOD Analytical, Chadds Ford, PA, United States), which was held at 50°C. Mobile phase A was 85:15:0.1 water:acetonitrile:acetic acid. Mobile phase B was 70:30:0.1 acetonitrile:methanol:acetic acid. Flow rate was 400 ml/min. Gradient elution was used; mobile phase percentage B and flow rate were varied as follows: 20% B at 0 min, ramp from 0 to 5 min to 40% B, ramp from 5 to 7 min to 55% B, ramp from 7 to 13 min to 64% B. From 13 to 19 min the column was flushed with 100% B at a flow rate of 550Turbo desolation gas was heated to 425°C at a flow rate of 6 L/min. Negative ion electrospray ionization tandem mass spectrometry with multiple reaction monitoring was used for detection. Quantification was done using Analyst 1.5.1 software comparing relative response ratios for each analyte/internal standard to standard curves for each analyte.

### Echocardiography Measurements

Non-invasive functional assessment was performed by transthoracic echocardiography using a Vevo 770 high-resolution imaging system with a 30 MHz transducer (RMV-707B; VisualSonics). Isoflurane (0.8% by anesthetic machine) was used to anesthetize the mice during the recordings. To assess the change in cardiac function, echocardiography was carried 24 h after LPS administration. Left ventricular end-systolic diameter (LVESD) and end-diastolic diameter (LVEDD) were obtained from M-mode images as well left atrial size was obtained by M-mode imaging in the parasternal long axis view. Systolic function was assessed by calculating ejection fraction (%EF) and fractional shortening (%FS) using the following equations %EF = (LVEDV-LVESV/LVEDV) × 100 and %FS = (LVEDD-LVESD/LVEDD) × 100. Tei index was calculated as [isovolumic contraction time (IVCT) + isovolumic relaxation time (IVRT)]/ejection time (ET). Diastolic function was assessed using pulsed-wave Doppler imaging as previously described (Akhnokh et al., 2016; Jamieson et al., 2017b) VisualSonics software was used for the qualitative and quantitative measurements.

### Inflammatory Response

Blood samples were collected and probed for TNFα and MCP-1 using ELISA kits (Abcam, Toronto, ON, Canada). Briefly, the sample was added into individual wells of a 96-well plate coated with a TNFα or MCP-1 mouse-specific antibody. After washing, wells were incubated with HRP-conjugated streptavidin, washed and incubated with substrate solution and measured spectrophotometrically at 450 nm. Increased color intensity occurred in a linear proportion to the amount of TNFα or MCP-1 in the samples. NF-kB DNA binding activity was measured using an ELISA kit from Active Motif (Carlsbad, CA, United States), which is based on the specific recognition of NF-kB response elements.

### Cell Culture

HL-1 cardiac cells were a kind gift from Dr. Claycomb (New Orleans, United States). Cells were cultivated in Claycomb media supplemented with glutamine and norepinephrine as described (Samokhvalov et al., 2012). HL-1 cells were maintained at 37°C in a humidified atmosphere of 5% CO2 and 95% air. Neonatal rat cardiomyocytes (NRCMs) were isolated from 2 to 3 day-old pups as previously described before (Samokhvalov et al., 2012) and were cultivated in DMEM medium supplemented with 10% FBS at 37°C in a humidified incubator maintaining 5% CO_2_ and 95% air. Cell viability was assessed using the CCK-8 viability kit (Sigma-Aldrich, Oakville, ON, Canada) based on the production of water-soluble formazan by dehydrogenases of viable cells. HL-1 cells were treated or co-treated with either 9,10-DiHOME, 12,13-DiHOME, 9,10-EpOME, 12,13-EpOME (0.01, 0.1, or 1 μM), and/or *t*AUCB (10 μM) to specifically inhibit sEH activity. 9,10-DiHOME, 12,13-DiHOME, 9,10-EpOME, and 12,13-EpOME were obtained from Cayman Chemical (Ann Arbor, MI, United States) and *t*AUCB was kindly provided by Dr. Bruce Hammock, UC, Davis, United States. Caspase-3/7 activity in cardiomyocytes was detected by using the Apo-ONE assay kit (Promega, Madison, WI, United States) according to manufacturer’s instructions. Aconitase 2 enzymatic activity as a marker of mitochondrial injury was assessed using a kit from (Abcam Toronto, ON, Canada).

### Assessments of Mitochondrial Function and Biogenesis

In order to test mitochondrial function we measured ATP levels using a luciferase-based method (Sigma-Aldrich, Oakville, ON, Canada). Mitochondrial respiration was measured in saponin (100 μg/ml) permeabilized HL-1 cells using Clark oxygen electrode connected to Oxygraph Plus recorder (Hansatech Instruments Ltd., Norfolk, England) (Akhnokh et al., 2016). Respiration rates were measured at 30°C before and after addition of 2 mM ADP where 5 mM malate and 10 mM glutamate were used as respiratory substrates. Respiratory control ratio (RCR) was calculated as the ratio between basal and ADP-stimulated respiration rates. Mitobiogenesis was evaluated using an ELISA kit (Abcam, Toronto, ON, Canada) based on simultaneous detection of SDH-A, a subunit of complex II (nDNA- encoded protein) and COX-1, a subunit of complex IV (mtDNA-encoded).

### Microscopic Analysis

HL-1 cells were plated in 6-well glass bottom plate and left until 80–90% confluency. Cells were loaded with 1 nM tetramethylrhodamine ethyl ester (TMRE) and 1 μM Hoechst 33342 trihydrochloride, 30 min prior to treatment. TMRE was used to reflect mitochondria and Hoechst 33342 to identify cell nuclei. Cells were treated with EpOMEs (0,10 nM or 100 nM) and DiHOMEs (0,10 nM or 100 nM) for 6 h. Images were taken by Zeiss Axio Observer Z1 inverted epifluorescence microscope, using 63X oil lens and maintained at 37°C and 5% CO_2_ throughout the experiment.

### Statistical Analysis

Data are presented as mean ± SEM. Statistical analysis was based on two-way ANOVA with Tukey’s *post hoc* test; *P* < 0.05 was considered statistically significant. All statistical analysis was performed using GraphPad Prism 7 software.

## Results

### sEH Null Mice Had Attenuated Inflammatory Responses Evoked by LPS

**FIGURE 1:**
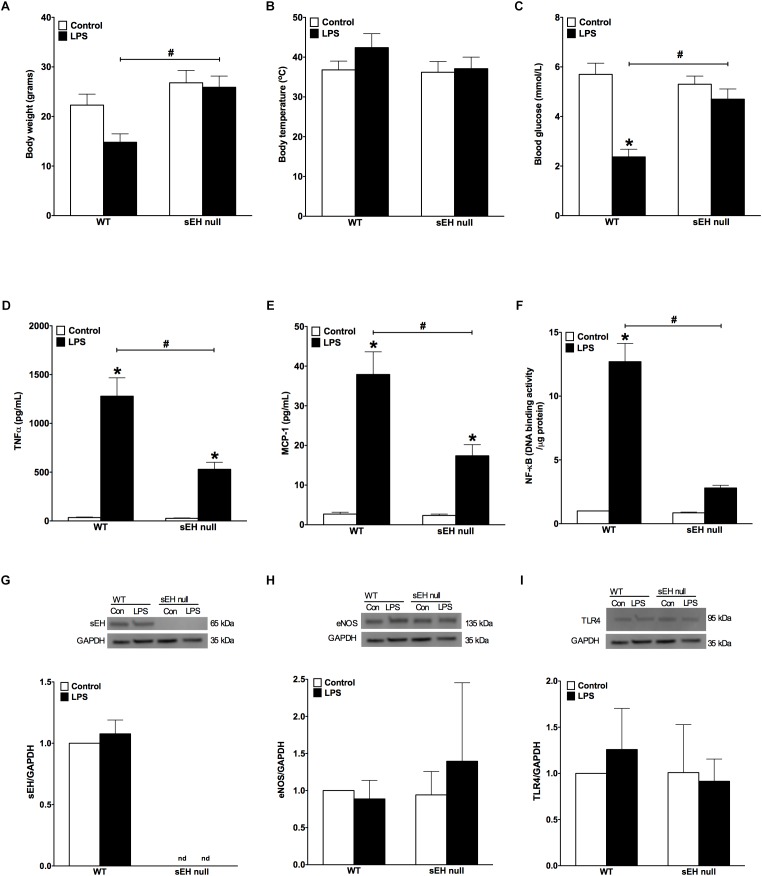
Changes to body weight, temperature, blood glucose levels and inflammatory responses following LPS administration in WT and sEH null mice. **(A)** Differences in body weight of WT and sEH null mice with or without injection of LPS, **(B)** differences in body temperature in WT and sEH null mice with or without injection of LPS, **(C)** comparing blood glucose levels for WT and sEH mice with or without injection of LPS, **(D)** serum levels of TNFα in WT and sEH null mice, **(E)** serum levels of MCP-1 in WT and sEH null mice, **(F)** DNA binding activity of NF-kB in hearts from WT and sEH null mice. Representative immunoblots and densitometric quantification of **(G)** sEH (60 kDa), **(H)** eNOS, (135 kDa), and **(I)** TLR4 (95 kDa) protein expression in hearts from WT and sEH null mice. All expression was normalized to GAPDH loading control. Data presented as mean ± SEM, *N* = 4–6. Statistics: Two-way ANOVA with Tukey’s *post hoc* test. ^∗^*p* < 0.05 vs. control, ^#^*p* < 0.05 vs. LPS treatment groups.

In order to assess whether sEH deficiency provides resistance, we induced acute inflammation in WT and sEH null mice using a clinically relevant model where LPS was administrated i.p. (10 mg/kg). Administration of LPS resulted in weight loss, increased body temperature and significantly reduced levels of blood glucose in WT mice (Figures 1A–C). The physiological changes we observed in WT mice following exposure to LPS reflect common responses that occur in acute murine sepsis such as fever, anorexia and subsequent hypoglycemia (Wisse et al., 2007; Yue et al., 2015). In contrast, sEH null mice had reduced physiological responses to LPS exposure compared to the WT mice (Figures 1A–C). These observations demonstrate that deficiency of sEH confers a high degree of physiological tolerance to LPS-induced acute inflammation. To determine whether sEH expression was altered following LPS injection, sEH protein expression was then assessed in WT and sEH null hearts. There were no significant changes in WT hearts exposed to LPS (Figure 1G) with no expression in either control or LPS groups in sEH null hearts as expected. In order to obtain insight into key proteins involved in immune responses we assessed hearts for expression of eNOS and TLR4 (Figures 1H, I). There were no significant differences between WT and sEH null mice in either control or LPS groups for eNOS and TLR4 proteins.

LPS-induced injury is largely mediated through robust activation of the pro-inflammatory response. Particularly, LPS-associated inflammation appears to be a driving force causing inflammatory-associated damage and dysfunction of the myocardium. First, we investigated if WT differed from sEH null mice in terms of overall inflammatory reactions developed in response to LPS administration. We found significantly lower levels of both TNFα and MCP-1 cytokines in blood of sEH null versus WT mice (Figures 1D,E), suggesting the inflammatory response was attenuated in sEH null mice. We next investigated the effect of sEH deficiency toward activation of a specific inflammatory response, NF-kB DNA binding activity. Consistent with the reduced inflammatory response, we found NF-κB DNA binding activity was significantly lower in myocardium of sEH null mice compared to WT (Figure 1F). Together, these data indicate that deficiency of sEH confers protection against inflammation occurring in response to LPS challenge.

### sEH Deficiency Attenuated Against LPS-Induced Cardiac Dysfunction

Development of LPS-induced inflammation is associated with multiple organ failure as a result of an uncontrollable innate immune response, which impacts cardiac function. We determined if sEH deficiency protects against cardiac dysfunction resulting from LPS-induced acute inflammation. Baseline heart function was similar between WT and sEH null mice; however, there was a decline in heart function in WT mice following 24 h LPS exposure (Table 1). No significant differences in heart rate were observed between WT and sEH null mice, suggesting any changes to LV function were not attributed to fluctuations in HR. Assessment of LV internal diameter during diastole and systole revealed LPS-mediated increases for WT hearts. The decreased ejection fraction and fractional shortening observed following LPS administration demonstrated a significant reduction of systolic function in WT mice (Table 1). Our previous observations demonstrated genetic deletion of sEH significantly attenuated the cardiac dysfunction following myocardial infarction (Akhnokh et al., 2016). Similarly, LPS-mediated cardiac dysfunction characterized by significantly decreased %EF, %FS, and increased LV volumes was primarily observed in WT mice with sEH mice exhibiting preserved systolic function 24 h post-LPS injection (Table 1).

**Table 1:** Echocardiography data demonstrated various changes between WT and sEH null mice when treated with LPS.

		WT		sEH null	
		
		Control	LPS	Control	LPS
HR, beats/min		484 ± 22	466 ± 7	517 ± 24	495 ± 40
**Wall measurements**					
	Corrected LV mass, mg	71.68 ± 5.97	80.34 ± 7.98	70.47 ± 2.65	84.95 ± 7.64
	IVS-diastole, mm	0.70 ± 0.04	0.71 ± 0.03	0.74 ± 0.04	0.77 ± 0.06
	IVS-systole, mm	1.18 ± 0.13	0.99 ± 0.08	1.07 ± 0.07	1.18 ± 0.12
	LVPW-diastole, mm	0.72 ± 0.05	0.67 ± 0.03	0.69 ± 0.03	0.77 ± 0.08
	LVPW-systole, mm	1.09 ± 0.09	0.92 ± 0.08	1.18 ± 0.03	1.18 ± 0.17
	LVID-diastole, mm	3.7 ± 0.2	4.1 ± 0.2	3.7 ± 0.1	3.9 ± 0.1
	LVID-systole, mm	2.3 ± 0.2	3.2 ± 0.3	2.3 ± 0.1	2.6 ± 0.3
**Cardiac function**					
	EF, %	72.46 ± 2.08	45.78 ± 6.25*	68.06 ± 1.96	61.83 ± 6.70
	FS, %	41.04 ± 1.67	22.85 ± 3.62*	37.35 ± 1.61	33.57 ± 5.19
	LVEDV, μl	55.44 ± 3.98	74.80 ± 7.78	57.84 ± 4.33	67.93 ± 4.97
	LVESV, μl	15.76 ± 2.28	41.10 ± 7.34*	18.49 ± 1.91	26.78 ± 6.17
	CO, ml/min	19.03 ± 0.47	15.66 ± 2.22	20.61 ± 2.31	20.21 ± 1.26
	SV, μl	39.68 ± 1.96	33.73 ± 4.88	39.35 ± 3.06	41.18 ± 2.21
**Doppler imaging**					
	IVRT, ms	13.1 ± 2.3	24.2 ± 1.1*	13.5 ± 0.7	19.0 ± 0.7
	IVCT, ms	10.6 ± 1.5	17.6 ± 2.1*	7.6 ± 0.8	16.0 ± 1.6*
	ET, ms	53.5 ± 3.1	43.1 ± 3.4	42.4 ± 2.6	45.6 ± 2.1
	Tei index	0.45 ± 0.06	1.01 ± 0.16*	0.50 ± 0.04	0.77 ± 0.03


*Data presented as mean ± SEM, N = 4–6, ^∗^p < 0.05 vs. control.*

### sEH Null Mice Are Resistant to Myocardial Oxidative Injury in Response to LPS

**FIGURE 2:**
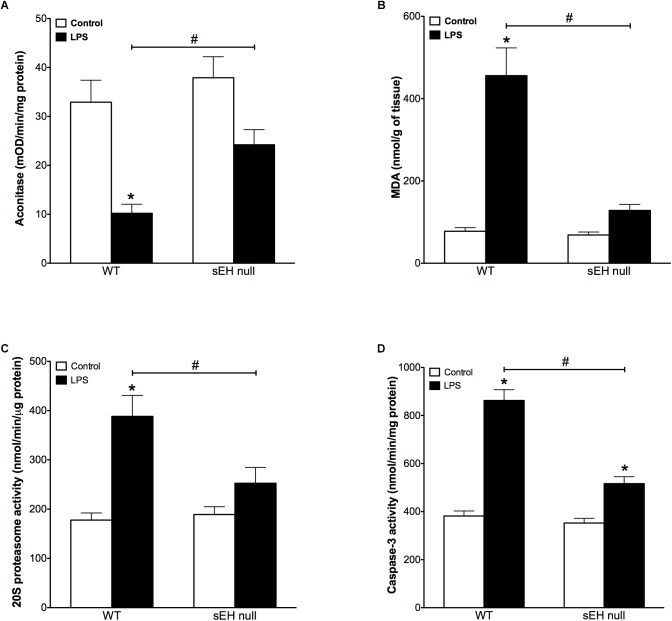
Aconitase levels are recovered in sEH null mice after injection of LPS whereas MDA, 20S proteasome and caspase-3 activity are reduced in sEH null mice post LPS treatment. **(A)** Aconitase activity, **(B)** heart malondialdehyde (MDA) levels, **(C)** 20s proteasome activity, and **(D)** cardiac caspase-3 activity in hearts from WT and sEH null mice. Data presented as mean ± SEM, *N* = 4–6. Statistics: Two-way ANOVA with Tukey’s *post hoc* test. ^∗^*p* < 0.05 vs. control, #*p* < 0.05 vs. LPS treatment groups.

Accumulation of oxidative injury in myocardium has been recognized as one of the key factors mediating cardiotoxicity of LPS (Suliman et al., 2004; Vanasco et al., 2008). Aconitase is an enzyme that catalyzes the reversible interconversion of citrate and isocitrate in the TCA cycle. Importantly, aconitase also stabilizes mtDNA thereby influencing mitochondrial gene expression. A decrease in aconitase activity is considered a marker of damaged mitochondrial structures. Our observations demonstrate that inflammation induced significantly stronger decrease in aconitase activity of WT mice than in those of sEH null mice (Figure 2A). In order to examine if sEH null mice are resistant to oxidative injury in the setting of LPS- induced inflammation, we employed a test measuring a total level of malondialdehyde (MDA). We found that LPS caused a dramatic increase in the levels of MDA in myocardium of WT mice indicative of extensive damages of cardiac structures. However, administration of LPS to sEH null mice did not result in much accumulation of myocardial MDA suggesting there was an adaptive event associated with sEH deficiency (Figure 2B). The accumulation of ubiquinated proteins triggers 20S proteasome activity to remove the targeted damaged proteins. As such, in accordance to our previously published studies, 20S proteasome activity can be utilized as a marker of unspecific cellular degenerative processes occurring in myocardium. Figure 2C clearly demonstrates that WT mice developed a sharp elevation in 20S proteasomal activity in myocardium after being challenged with LPS. Conversely, sEH null mice displayed significantly less activation providing further evidence of cardioprotection associated with deficiency of sEH (Figure 2C). Finally, we measured activity of caspase 3/7 (a marker of apoptosis) in myocardium of WT and sEH null mice. These data provide indirect evidence of increased activation of apoptotic responses in the myocardium of WT mice compared to sEH null animals (Figure 2D).

### Deletion of sEH Preserved Mitochondrial Function Following LPS Treatment

**FIGURE 3:**
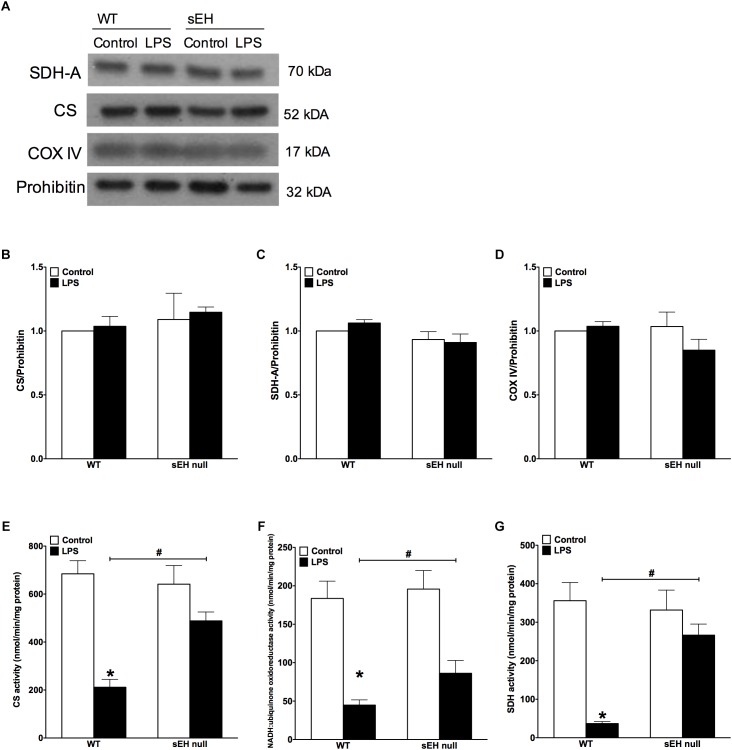
Key mitochondrial oxidative metabolism enzyme levels had no significant change but activities in LPS treated WT mice is significantly reduced. **(A)** Representative immunoblots of succinate dehydrogenase (SDH-A 70 kDa), citrate synthetase (CS 52 kDa), cytochrome c oxidase IV (COX IV 17 kDa), and loading control prohibitin (32 kDa). Densitometric quantification of **(B)** CS, **(C)** SDH-A, **(D)** COX IV, and **(E)** CS activity, **(F)** NADH:ubiquinone oxidoreductase activity, and **(G)** succinate dehydrogenase activity in hearts from WT and sEH null mice. Data presented as mean ± SEM, *N* = 5. Statistics: Two-way ANOVA with Tukey’s *post hoc* test. ^∗^*p* < 0.05 vs. control, ^#^*p* < 0.05 vs. WT LPS treatment groups.

**FIGURE 4:**
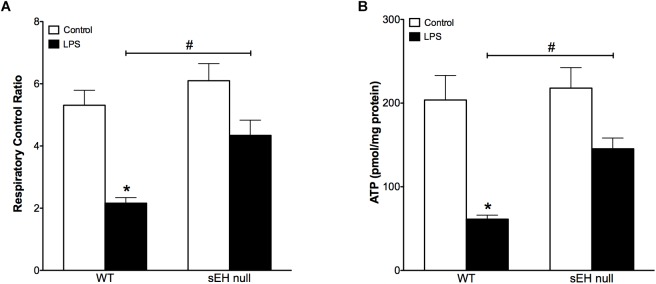
Reduced respiratory control ratio and myocardial ATP levels indicate decreased mitochondrial efficiency and function which was preserved in sEH null mouse hearts. **(A)** The ratio between basal mitochondrial respiration rate and ADP-stimulated state RCR is reduced in WT mice injected with LPS, a significant recovery in RCR was observed in sEH null mice. **(B)** Amount of ATP is significantly reduced in WT LPS treated mice and a recovery was observed for sEH null mice. Data presented as mean ± SEM, *N* = 4. Two-way ANOVA with Tukey’s *post hoc* test. ^∗^*p* < 0.05 vs. control, ^#^*p* < 0.05 vs. WT LPS treatment groups.

Based on our previous data demonstrating adverse effects of LPS toward cardiac mitochondria, it was important to further evaluate alterations in mitochondrial function and whether sEH deficiency confers mitochondrial protection against LPS insult (Samokhvalov et al., 2015). Accordingly, we first measured protein content and then activity of key enzymes of mitochondrial oxidative metabolism such as citrate synthase, complex I and complex II. No significant alterations in the expression of cardiac mitochondrial proteins was observed in hearts from WT or sEH null mice, suggesting overall mitochondrial content was preserved (Figures 3A–D). However, LPS WT animals had significantly lower catalytic activity in the tested enzymes indicative of an overall decrease in mitochondrial function (Figures 3E–G). Remarkably, administration of LPS did not produce severe inhibition of enzymatic activities in the hearts of sEH null mice providing further evidence of cardioprotection was associated with preservation of mitochondrial function. Analysis of mitochondrial O_2_ consumption is the classical approach to characterize mitochondrial function by determining the respiratory rates during mitochondrial respiratory states such as basal state (resting or controlled respiration) and in ADP-stimulated state (active respiration, the maximal physiological rate of O_2_ uptake, and ATP synthesis). The ratio between the states represents a level of physiological efficiency of mitochondria and expressed as RCR. As shown in Figure 4A, we observed a significant decline in RCR of cardiac mitochondria isolated from LPS-exposed WT mice that was significantly attenuated in sEH null mice. Noteworthy, malate and glutamate were used as respiratory substrates. Considering the fact that oxidation of malate-glutamate is controlled exclusively by complex I, our observations tentatively suggest there was an impairment in complex I causing further limitation of the entire respiratory rates. This finding is indirectly supported by a previous report suggesting inactivation of respiratory activity following acute LPS exposure compromises optimal mitochondrial function (Frisard et al., 2015). Myocardial ATP content is a classical marker of mitochondrial function. LPS exposure significantly decreased ATP content in the hearts of WT animals (Figure 4B). While a moderate drop in ATP levels was observed in sEH null mice following LPS exposure, ATP levels were significantly better in sEH null hearts compared to WT mice (Figure 4B). All together, these data directly suggests a metabolic collapse of the heart developed in WT mice exposed to LPS, which can be considered as a beginning of heart failure. In striking contrast, mitochondria isolated from the hearts of sEH null animals exhibited preserved hallmarks of oxidative metabolism indicative of adaptive cardioprotection originating most likely within mitochondria.

### Cardiac Oxylipin Levels Following Acute Inflammation in WT and sEH Null Mice

We quantified metabolites of arachidonic acid, linoleic acid, EPA, and DHA derived epoxides and diols in myocardium from WT and sEH null mice challenged with or without LPS utilizing LC-MS/MS (Table 2). Genetic deletion of sEH resulted in moderate changes in epoxides derived from linoleic acid (12,13-EpOME and 9,10-EpOME), arachidonic acid, EPA, and DHA metabolites, generated via catalytic pathways mediated by COX, LOX, and CYP450. Following 24 h exposure to LPS there was an expected increase in pro-inflammatory metabolites in WT mice. This was marked by increased production of COX metabolites of AA (prostanoids), 6-keto-PGF1α, PGF2α, PGE2, and PGD2, reflecting a pro-inflammatory response. PGE2 is a predominant pro-inflammatory prostanoid that enhances edema formation and leukocyte infiltration by promoting blood flow, while PGD2 is a major product of mast cells, contributing to inflammation. The changes were attenuated in sEH null mice following LPS treatment supporting the anti-inflammatory effect. There were no statistically significant alterations in LOX-dependent metabolites of AA (5-, 8-, 12-, and 15-HETE). However, there were trends suggesting increases in LA [9- and 13-hydroxyoctadecadienoic acid (HODE)] metabolites in WT mice following LPS exposure, which were absent in sEH null mice.

**Table 2:** Cardiac oxylipin levels in WT or sEH deficient mice following LPS treatment.

	WT		sEH null	
		
	Control	LPS	Control	LPS
**CYP epoxygenase dependent metabolism**				
12,13-EpOME	29.25 ± 3.34	50.35 ± 7.99	56.95 ± 8.89	47.2 ± 7.03
9,10-EpOME	32.55 ± 3.63	59.07 ± 8.94	52.72 ± 8.52	45.2 ± 3.64
14,15-EET	2.97 ± 0.24	3.25 ± 0.5	3.2 ± 0.64	3.31 ± 0.48
11,12-EET	2.44 ± 0.24	2.74 ± 0.18	2.99 ± 0.39	2.96 ± 0.37
8,9-EET	3.25 ± 0.44	4.09 ± 0.5	3.69 ± 0.75	3.6 ± 0.52
5,6-EET	ND	ND	ND	ND
17,18-EpETE	1.19 ± 0.17	0.83 ± 0.067	2.6 ± 0.29	1.98 ± 0.95
19,20-EpDPE	72.67 ± 15.95	81.2 ± 20.89	65.42 ± 23.01	60.75 ± 11.18
**sEH dependent metabolism**				
12,13-DiHOME	11.58 ± 1.88	43.27 ± 2.9*	4.1 ± 0.77	3.58 ± 0.79#
9,10-DiHOME	6.28 ± 1.15	19.6 ± 0.68*	6.84 ± 1.07	7.33 ± 1.24#
14,15-DHET	0.79 ± 0.15	1.15 ± 0.23	0.24 ± 0.05	0.22 ± 0.038#
11,12-DHET	0.4 ± 0.039	0.76 ± 0.14*	0.26 ± 0.043	0.25 ± 0.026#
8,9-DHET	0.37 ± 0.039	0.96 ± 0.11*	0.28 ± 0.059	0.23 ± 0.04#
5,6-DHET	0.47 ± 0.049	0.62 ± 0.047	0.29 ± 0.087	0.35 ± 0.035#
19,20-DiHDPA	3.84 ± 0.95	1.70 ± 0.23	9.15 ± 1.37	1.78 ± 0.28
**CYP hydrolase dependent metabolism**				
20-HETE	ND	ND	ND	ND
**LOX-dependent metabolism**				
15-HETE	6.24 ± 1.19	6.97 ± 0.75	5.52 ± 1.15	6.67 ± 0.82
12-HETE	13.89 ± 2.53	172.35 ± 145.55	13.76 ± 2.83	11.01 ± 1.82
11-HETE	2.71 ± 0.47	4.05 ± 0.45	2.45 ± 0.36	2.62 ± 0.2
8-HETE	50.57 ± 6.38	78.67 ± 23.16	47.35 ± 8.47	50.35 ± 7.95
5-HETE	3.39 ± 0.51	3.6 ± 0.5	3.35 ± 1.07	3.69 ± 0.49
9-HODE	38.45 ± 8.85	65.025 ± 7.89	42.8 ± 9.89	41.8 ± 5.2
13-HODE	163.35 ± 39.12	283 ± 30.7	165.17 ± 35.03	171.2 ± 21.9
**COX-dependent metabolism**				
6-keto-PGF1α	4.39 ± 1.046	9.89 ± 1.86*	3.86 ± 0.59	4.39 ± 0.3#
PGF2α	0.96 ± 0.25	1.65 ± 0.35	0.81 ± 0.16	0.63 ± 0.024
PGE2	0.61 ± 0.07	1.21 ± 0.24	0.8 ± 0.34	0.99 ± 0.25
PGD2	0.33 ± 0.05	1.23 ± 0.28*	0.39 ± 0.09	0.47 ± 0.1#


*Values (pg/ml) are represented as mean ± SEM, N = 4 independent experiments, ^∗^p < 0.05 vs. WT control, ^#^p < 0.05 vs. treatment group vs. WT-LPS.*

CYP epoxygenase metabolites of LA (EpOMEs), AA (EETs), EPA (17,18-EpETE), and DHA (19,20-EpDPEs) were found in significant amounts in control hearts (Table 2), which were higher in sEH null mice compared to WT. While LPS exposure did not significantly increase levels of 9,10-, 12,13-EpOME, and 8,9-, 11,12-, 14,15-EET, 17,18-EpETE, or 19,20-EpDPE, they were elevated in WT mice following LPS exposure. There were no changes in EpOME, EET, EpETE, or EpDPE levels observed in sEH null mice following LPS exposure. There was a significant increase in corresponding sEH-dependent diol products in WT hearts, which were significantly lower in sEH null hearts.

### DiHOMEs Cause an Inflammatory Response and Cytotoxicity in Cardiac Cells

**FIGURE 5:**
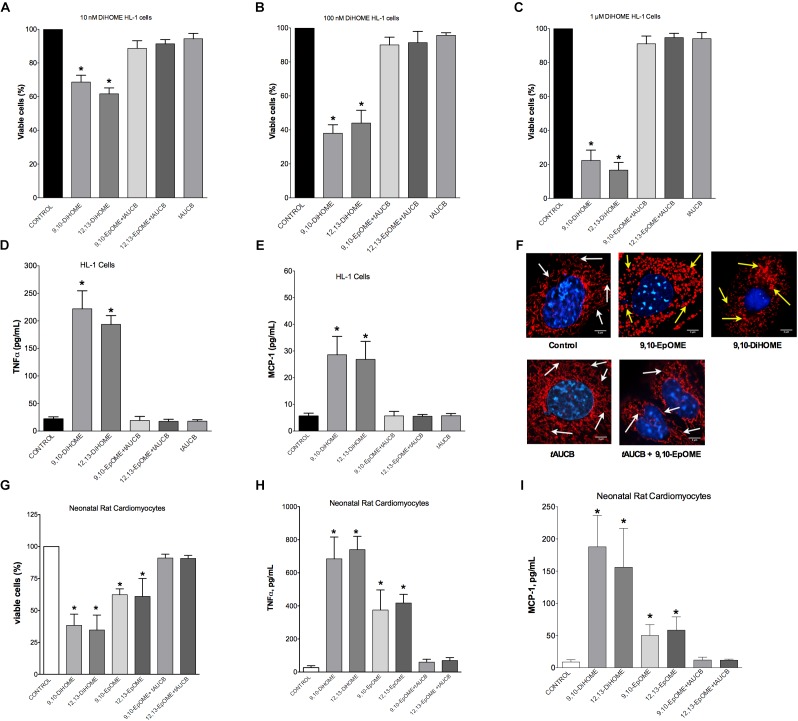
Concentration-response in HL-1 cells and neonatal cardiomyocytes following treatment with DiHOMEs. Hl-1 cell viability following 24 h treatment with **(A)** 10 nM of DiHOMEs, 10 μM *t*AUCB or 10 nM 9,10-EpOME with *t*AUCB, **(B)** 100 nM of DiHOMEs, 10 μM *t*AUCB or 10 nM 9,10-EpOME with *t*AUCB, **(C)** 1 μM of DiHOMEs, 10 μM *t*AUCB or 10 nM 9,10-EpOME with *t*AUCB, **(D)** TNFα levels secreted by HL-1 cells treated with 100 nM of DiHOMEs, 10 μM *t*AUCB or 10 nM 9,10-EpOME with *t*AUCB, **(E)** MCP-1 levels secreted by HL-1 cells treated 100nM of DiHOMEs, 10 μM *t*AUCB or 10 nM 9,10-EpOME with *t*AUCB. **(F)** Representative images of mitochondrial morphology in HL-1 cells following treatment for 6 h with 10 nM 9,10-EpOME, 10 nM 9,10-DiHOME, 10 μM *t*AUCB or 9,10-EpOME with *t*AUCB. Mitochondrial morphology, filamentous and tubular shape, of control cells are highlighted by white arrows. In contrast, treated cells exhibit significant punctate and fragmented mitochondrial morphology that are highlighted by yellow arrows. **(G)** Cell viability, **(H)** TNFα levels and **(I)** MCP-1 levels secreted from neonatal rat cardiomyocytes following 24 h treatment with either 100 nM 9,10-DiHOME, 100 nM 12,13-DiHOME, 100 nM 9,10-EpOME, 10 nM 12,13-EpOME, 100 nM 9,10-EpOME with 10 μM *t*AUCB or 100 nM 12,13-EpOME with 10 μM *t*AUCB. Data presented as mean ± SEM, *N* = 4. Statistics: One-way ANOVA with Bonferroni *post hoc* test. ^∗^*p* < 0.05 vs. control.

Evidence from literature indicates both beneficial and detrimental effects of EpOMEs and DiHOMEs within the cardiovascular system. As we observed large alterations in the DiHOME metabolites following LPS exposure in WT hearts, we further explored their effects in HL-1 cardiac cells and NRCMs. Treatment with 9,10- or 12,13-DiHOME resulted in a concentration-dependent decline in cell viability, beginning as low as 10 nM (Figures 5A–C). Conversely, HL-1 cells treated with different concentrations of 9,10-, or 12,13-EpOME in the presence of a sEH inhibitor (tAUCB) to prevent the conversion to DiHOMEs failed to cause any alteration in cell viability. In order to determine if DiHOMEs triggered a release of major pro-inflammatory cytokines such as TNFα and MCP-1 we assessed the response in HL-1 cells. Consistent, both 9,10- and 12,13-DiHOME caused massive release of TNFα and MCP-1 from HL-1 cells (Figures 5D,E). Together, these observations support a concept where accumulation of DiHOMEs may orchestrate an inflammatory cascade.

Epifluorescence microscopy images were used to assess alterations to mitochondrial morphology in HL-1 cells treated with 10 nM 9,10-DiHOME, 9,10-EpOME, or 9,10-EpOME with *t*AUCB for 6 h (Figure 5F). Our data suggest that treatment with 10 nM 9,10-DiHOME and 9,10-EpOME alone resulted in a pronounced degradation of mitochondria, as reflected by the markedly punctuated appearance and reduced size compared to the threadlike appearance in controls (Figure 5F). However, inhibition of sEH with *t*AUCB prevented the effect of 9,10-EpOME suggesting the toxic effect mitochondrial morphology was attributable to the DiHOME metabolite. Similar to HL-1 cells, NRCM treated with 9,10- or 12,13-DiHOME (100 nM) for 24 h resulted in a decline in cell viability, release of TNFα and MCP-1 (Figures 5G–I). Interestingly, when NRCM were 9,10- or 12,13-EpOME (100 nM) alone for 24 h there was less but still a significant loss in cell viability and release of TNFα and MCP-1. Importantly, the co-treatment of 9,10- or 12,13-EpOME (100 nM) with the sEHi (*t*AUCB) attenuated the adverse effects, again suggesting the DiHOMEs are responsible for the observed responses.

### HL-1 Cardiac Cells Exposed to DiHOMEs Revealed Profound Mitochondrial Dysfunction

**FIGURE 6:**
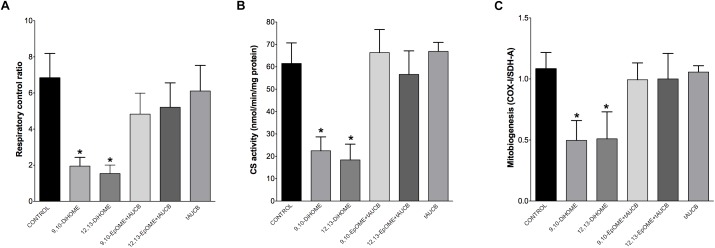
HL-1 cells treated with DiHOMEs show significant reductions in respiratory control ratio, mitobiogenesis and citrate synthase activity. **(A)** Significantly reduced respiratory control ratio (RCR) is seen in HL-1 cells treated with 100 nM of DiHOMEs, 10 μM *t*AUCB or 100 nM 9,10-EpOME with *t*AUCB, **(B)** citrate synthase (CS) activity in HL-1 cells treated with 100nM of DiHOMEs, 10 μM *t*AUCB or 100 nM 9,10-EpOME with *t*AUCB. And **(C)** mitobiogenesis in HL-1 cells treated with 100 nM of DiHOMEs, 10 μM *t*AUCB or 100 nM 9,10-EpOME with *t*AUCB. Data presented as mean ± SEM, *N* = 4. Statistics: One-way ANOVA with Bonferroni *post hoc* test. ^∗^*p* < 0.05 vs. control.

The most essential component of mitochondrial function is respiration coupled with generation of ATP also referred as oxidative phosphorylation. The ratio between oxygen consumption by mitochondria in basal and ADP-stimulated states indicates respiratory control ratio, which reflects bioenergetic efficiency of mitochondria. We measured mitochondrial respiration in permeabilized HL-1 cells to characterize the effects of DiHOMEs on mitochondrial function. Treatment with 9,10- or 12,13-DiHOME resulted in a strong decrease in mitochondrial RCR, suggesting there was an overall collapse in mitochondrial function, while treatment with 9,10- or 12,13-EpOME with tAUCB did not impact RCR (Figure 6A). This finding is supported by another observation where exposure to only 9,10- or 12,13-DiHOME resulted in a strong decrease in CS activity in HL-1 cardiac cells (Figure 6B). Generation of new pool of mitochondria appears to be an important physiological strategy developed to withstand stress conditions. Numerous studies have demonstrated that disruption of mitobiogenesis causes extensive cardiac dysfunction. HL-1 cardiac cells exposed to DiHOMEs displayed a rapid decline in overall rate of mitobiogenesis, which was detected based on the ratio between expression of the mitochondrial proteins COX-1 (mtDNA-encoded) and SDH-A (nDNA-encoded) measured simultaneously. This observation suggests that DiHOMEs trigger a negative impact toward mitobiogenesis (Figure 6C).

## Discussion

Excessive inflammatory responses can cause a number of injurious events at both the systemic and cellular levels, such as inhibiting mitochondrial function, activating the immune system and nonspecific degenerative processes. Cardiovascular systems are highly susceptible to deleterious effects of LPS; however, the specific mechanisms are complex and poorly understood. Although several trials have been attempted to protect the heart against LPS-induced injury, there remain no definitive effective therapies to prevent and/or limit injury (Landesberg et al., 2012; Wang et al., 2016; Zhang et al., 2017; Chen et al., 2018; Wei et al., 2018). Our understanding about the role of inflammatory mediators have in regulating injury is important for developing novel diagnostic and therapeutic approaches in sepsis and endotoxemia. In the present study we investigated the protective effect of sEH deficiency toward mitochondrial and cardiac function following LPS exposure. We have previously demonstrated that sEH deficiency is effective in protecting cardiac cells against LPS-induced injury (Samokhvalov et al., 2015). Furthermore, a recent study showed that sEH ablation attenuates the pulmonary inflammatory response in mice challenged with LPS (Wepler et al., 2016). Here, we used a mouse model of sEH deletion to demonstrate cardiac function is maintained *in vivo* following LPS-induced acute inflammation.

In our model, the acute exposure to LPS caused pronounced cardiac injury highlighted by a robust pro-inflammatory response indicated by elevated serum TNFα and MCP-1 cytokine levels as well as increased NF-kB DNA binding activity without any change in cardiac TLR4 or sEH protein expression. In left ventricular tissue, LPS exposure induced oxidative stress and amplified protein degradation as illustrated by elevated 20S proteasome activity, eventually leading to increased activity of the apoptotic enzyme caspase-3 and cell injury. Enhanced release of pro-inflammatory cytokines from the heart causes recruitment of mononuclear cells, which has been shown to assist in the development of cardiovascular disease (Anker and von Haehling, 2004; Zernecke et al., 2008). The therapeutic potential for limiting inflammatory-induced migration of immune cells in myocardium appears to be tremendous. Our findings illustrate that sEH deficiency reduced the serum level of pro-inflammatory response and limited cell injury in response to LPS administration. Together, the data suggest that deletion or inhibition of sEH correlates with lower rates in migration of mononuclear cells thereby limiting inflammatory associated cardiac dysfunction.

Numerous studies demonstrate that LPS is capable of damaging mitochondria by mechanisms such as oxidizing mitochondrial DNA, decreasing ATP production and increasing ROS generation, all of which contribute to cardiac dysfunction (Suliman et al., 2004; Vanasco et al., 2012). Further reports demonstrate these alterations may occur in a tissue specific manner, for example, mitochondrial oxygen consumption during state 3 respiration was reduced in cardiac and skeletal muscle in a rat model of acute inflammation (Rozenberg et al., 2006; Vanasco et al., 2008, 2012). The acute effect of LPS exposure reflect cellular responses to physiological cues that lead to mitochondrial uncoupling, altered respiration and a switch to a more rapidly metabolized substrate for ATP production (e.g., glucose) (Zhang et al., 2014; Frisard et al., 2015). Furthermore, the subsequent increased ROS production may stem from increased mitochondrial proton leak and uncoupling through either UCP3 dependent or independent mechanisms resulting in decreased mitochondrial efficiency directly leading to cardiovascular collapse (Frisard et al., 2015). The present study demonstrates LPS triggered mitochondrial damage and impaired the catalytic activity of key markers of oxidative phosphorylation resulting in a decline in mitochondrial respiration and ATP content, indicative of impaired mitochondrial function. Considering we did not observe changes in mitochondrial protein levels but decreased function in WT mice compared to sEH null, our data suggest the pool of mitochondria found in sEH null hearts was healthier. As such, the adverse effects in WT mice may be attributed to either direct impact on function and/or impeded mitochondrial quality control mechanisms.

In response to inflammation and cell injury succinate can accumulate in the mitochondria, inhibiting complex I activity and inducing generation of ROS. This results in the lower rates of electron transfer through complex I observed in acute inflammation, which critically restricts ATP synthesis and results in a situation further exacerbating organ damage through a self-sustaining process. Thus, acute inflammation provokes a suppression of cardiac mitochondrial function accompanied by increased accumulation of injury (Galkin et al., 2009; Navarro et al., 2009, 2010). Compounding all factors presented, the synergetic magnitude of these events instigated cardiac collapse. Ultimately, these changes contributed to the pronounced dilation of the myocardium accompanied by drastic decrease in ejection fraction we observed in the WT mice, indicative of a progression to early heart failure. In striking contrast, sEH null animals presented conserved oxidative metabolism suggesting adaptive cardioprotection originating most likely in the mitochondria. These data indicate that deficiency of sEH confers protection against cardiac inflammation and mitochondrial damage upon LPS challenge. Intriguingly, these data are associated with fundamental changes in the LA-derived epoxides.

The anti-inflammatory effects of epoxides have been extensively studied since it was discovered inflammation plays a critical role in cardiovascular diseases. A tight functional connection between inflammation and epoxygenase enzymes has been established (Dennis and Norris, 2015). The anti-inflammatory actions of epoxides appear to be mediated through suppression of NF-kB activation. Activated NF-kB is a critical signaling molecule for the induction of numerous inflammatory mediators in all cells types including cardiomyocytes. Previously, we demonstrated that EETs effectively inhibit the activation of inflammatory reactions in cardiac cells in response to LPS (Samokhvalov et al., 2014). An important outcome of this study proposes that protective effects of sEH deficiency can be explained by an increased level of endogenously produced EETs as their metabolic degradation would be strongly suppressed. These findings have provided the framework for the notion sEH is a promising therapeutic target for the treatment of cardiac dysfunction associated with upregulated inflammatory response in the setting of LPS-induced acute inflammation. We and others have shown sEH deficiency and EETs, particularly 11,12-EET, are cardioprotective in models of cardiovascular injury and aging (Batchu et al., 2011; Akhnokh et al., 2016; Jamieson et al., 2017a). Moreover, sEH deficiency in pulmonary LPS-induced toxicity was associated with improved recovery and increased levels of EETs (Wepler et al., 2016). There is convincing evidence in the literature that EETs suppress NF-kB-mediated induction and the subsequent pro-inflammatory response through inhibition of IKK complex activity (Node et al., 1999; Deng et al., 2010; Zhao et al., 2012). Interestingly, in this model we saw no change in overall EET levels 24 h following LPS administration in either WT or sEH null mice. However, DHET levels were significantly reduced, suggesting that sEH deficiency lengthens the half-life of the EETs rather than increasing basal levels. The body may also compensate to the genetic ablation by reducing the production of EETs to preserve homeostasis, resulting in overall preserved EET levels.

Importantly, the acute LPS challenge markedly altered the cardiac oxylipin profile in the hearts, which included changes to various different metabolites. While this suggests multiple lipid mediators may be involved in effects we observed following LPS administration, the current study focused on the effect of DiHOMEs. Lipid mediators generated from eicosanoid metabolism have been demonstrated to modulate functional activity of mitochondria. Several models show pathological concentrations of LA uncouple oxidative phosphorylation, induce mitochondrial permeability transition, and activate processes leading to cell death (Schonfeld and Bohnensack, 1997; Moran et al., 2000, 2001; Schonfeld et al., 2000; Konkel and Schunck, 2011). This is supported by studies demonstrating pathological concentrations of 12,13-EpOMEs and 12,13-DiHOMEs are toxic to cardiac cells (Moran et al., 2000, 2001; Konkel and Schunck, 2011). Conversely, several studies suggest EpOMEs may be cardioprotective via maintenance of mitochondrial function (Moghaddam et al., 1997; Nowak et al., 2004; Hou et al., 2012). Although the specific roles of EpOMEs in inflammation and cellular homeostasis remain poorly understood, evidence indicates many of the cytotoxic effects attributed to EpOMEs are in fact caused by their secondary metabolites DiHOMEs. The accumulation of DiHOMEs in the heart is associated with impaired cardiac function, including that resulting from LPS-induced endotoxemic shock (Edin et al., 2011; Chaudhary K.R. et al., 2013). Therefore, DiHOMEs may be considered the crucial metabolites mediating the toxicity of LA epoxides (Moghaddam et al., 1997; Zeldin, 2001; Zheng et al., 2001; Fleming, 2014). Consistent with previous reports, 24 h after LPS administration the levels of DiHOMEs were elevated in left ventricular tissue revealing activation of the CYP P450 epoxygenase-sEH metabolic pathway during LPS-induced toxicity (Schmelzer et al., 2005; Tao et al., 2016). In contrast, deletion of sEH significantly attenuated DiHOME accumulation in left ventricular tissue following LPS administration, increasing the levels of EpOME relative to DiHOME production. Data from our cell studies indicated inhibition of EpOME metabolism with an sEH inhibitor prevented cytotoxic effects suggesting the DiHOME metabolite was causing the adverse response. Taken together, these data indicate sEH deletion serves as a twofold cardioprotective strategy to decrease DiHOME production while simultaneously preserve EET levels, ultimately acting to protect cardiac mitochondria and thus maintain overall cardiac function. In summary, our results demonstrate that accumulation of DiHOMEs correlates with LPS-induced cardiac toxicity and markedly reduces mitochondrial function and cell survival. However, preventing the metabolism of the EpOMEs in cardiac tissue through sEH deficiency preserves a pool of healthy mitochondria thereby limiting LPS-induced cytotoxicity and ultimately preserving cardiac function.

## Author Contributions

VS performed and planned the experiments, data analyses and writing of the manuscript. KLJ was involved with echocardiography, data analysis and writing the manuscript. AD and TL were involved in the experiments, data analysis and writing of the manuscript. HK-B performed immunoblot experiments and data analyses. ME, FL, and DZ performed the LC/MS metabolite profile and scientific evaluation. JMS is the primary investigator. JMS was involved in the planning and experimental design, data analyses and writing of the manuscript.

## Conflict of Interest Statement

The authors declare that the research was conducted in the absence of any commercial or financial relationships that could be construed as a potential conflict of interest.
